# Association Between Serum Ferritin and Hemoglobin Levels in Pregnant Women: Implications for Early Iron Deficiency Screening

**DOI:** 10.7759/cureus.114043

**Published:** 2026-08-06

**Authors:** Doaa Ahmed, Rawaal Amin, Vidya Raman, Madina Hashmi, Mohammad Ghaith Hulo, Baraa Albousen, Aleena Hameed, Kaushalendra Mani Tripathi, Nadia Naureen, Laraib Tariq, Ghosoun Ibrahem Atoom

**Affiliations:** 1 Obstetrics and Gynecology, Alexandria University, Alexandria, EGY; 2 Obstetrics and Gynecology, Guy's and St Thomas' NHS Foundation Trust, London, GBR; 3 Gynecology, Sandeman Provincial Hospital, Quetta, PAK; 4 Obstetrics and Gynecology, Osmania Medical College, Hyderabad, IND; 5 Obstetrics and Gynecology, Noble’s Hospital, Braddan, IMN; 6 Obstetrics and Gynecology, Wrexham Maelor Hospital, Wrexham, GBR; 7 Medicine, University of Aleppo, Aleppo, SYR; 8 Medicine, Pilgrim Hospital, Boston, GBR; 9 Obstetrics and Gynecology, University of Aleppo, Aleppo, SYR; 10 Obstetrics and Gynecology, Glan Clwyd Hospital, Rhyl, GBR; 11 Obstetrics and Gynecology, Russells Hall Hospital, Dudley Group NHS Foundation Trust, West Midlands, GBR; 12 Internal Medicine, Smt. N.H.L. Municipal Medical College, Ahmedabad, IND; 13 Medicine, White River Medical Center, White River Health, Batesville, USA; 14 Obstetrics and Gynecology, National Guards Hospital, Madinah, SAU; 15 Psychiatry and Behavioral Sciences, CMH Lahore Medical College and Institute of Dentistry, Lahore, PAK; 16 Medicine, Yarmouk University, Irbid, JOR

**Keywords:** anemia, hemoglobin, iron deficiency, pregnancy, serum ferritin

## Abstract

Background: Iron deficiency is the leading cause of anemia during pregnancy, and serum ferritin is the most widely used biochemical marker of body iron stores. However, population-specific evidence describing the relationship between serum ferritin concentrations and hemoglobin levels remains limited.

Purpose: This study aimed to assess the relationship between serum ferritin and hemoglobin levels among pregnant women attending antenatal care.

Methods: A cross-sectional, laboratory-based study was conducted among 522 pregnant women in their second and third trimesters. Serum ferritin concentrations were measured using the hospital's standardized laboratory procedures. Iron deficiency was defined as a serum ferritin concentration <30 µg/L, and anemia was defined as a hemoglobin concentration <11 g/dL. Statistical analyses included descriptive statistics, independent-samples Student's t-tests, chi-square tests, binary logistic regression, and receiver operating characteristic (ROC) curve analysis.

Findings: Iron deficiency was identified in 209 participants (40.0%), and anemia was present in 235 participants (45.0%). Serum ferritin concentrations and mean corpuscular volume (MCV) were significantly lower in women with anemia than in those without anemia (p < 0.001). Serum ferritin was independently associated with anemia (OR = 0.95, p < 0.001). Notably, 35 women without anemia (12.2%) had low serum ferritin concentrations, indicating subclinical iron deficiency. ROC curve analysis demonstrated good discriminative performance, with serum ferritin showing greater diagnostic accuracy than MCV. The optimal serum ferritin cutoff was 17.0 µg/L, with a sensitivity of 81% and a specificity of 83%.

Conclusion: Pregnant women with anemia had significantly lower serum ferritin concentrations than those without anemia, and a substantial proportion of women with normal hemoglobin levels had depleted iron stores. These findings support the potential value of serum ferritin as an adjunctive marker for assessing iron status during pregnancy. However, prospective studies are needed to further evaluate its clinical utility.

## Introduction

Pregnancy-related anemia remains a significant global health problem, particularly in low- and middle-income countries, because it is associated with poor maternal and neonatal outcomes and an increased risk of maternal and neonatal morbidity and mortality [1,2]. According to the World Health Organization, anemia during pregnancy is defined as a hemoglobin level below 11 g/dL, with iron deficiency representing the most common underlying pathophysiological mechanism [3,4]. Pregnant women are particularly susceptible because of the increased iron requirements needed to support maternal blood volume expansion, placental growth, and fetal development [5]. In Pakistan, anemia during pregnancy remains a major public health concern. Previous studies conducted in Pakistan have reported a high prevalence of anemia among pregnant women, while national survey data have also demonstrated a substantial burden of anemia among women of reproductive age [6,7]. These findings underscore the importance of improving iron status before and during pregnancy. Despite this high disease burden, evidence describing the relationship between serum ferritin and hemoglobin concentrations among pregnant women in Pakistan remains limited [6,7].

Serum ferritin is the most reliable biochemical indicator of body iron stores and is widely used to assess iron deficiency [8]. A low serum ferritin concentration typically indicates depleted iron stores, which may be reflected by a corresponding reduction in hemoglobin levels [9]. However, ferritin is also an acute-phase reactant, and its concentration may be elevated in the presence of inflammation, infection, or chronic disease, thereby complicating its interpretation [10].

Several studies have reported a positive correlation between low serum ferritin and low hemoglobin concentrations in pregnant women, suggesting that iron depletion is a major contributor to the development of gestational anemia [11,12]. However, the strength and consistency of this association vary across populations because of differences in nutritional status, gestational age, socioeconomic conditions, and the presence of infection [13]. Furthermore, physiological inflammatory changes during pregnancy may influence serum ferritin concentrations because ferritin functions as an acute-phase reactant. Therefore, normal serum ferritin levels do not always exclude iron deficiency, and anemia during pregnancy may also result from other causes, such as hemoglobinopathies or anemia of inflammation [14].

Mean corpuscular volume (MCV) is typically reduced in iron deficiency and iron-deficiency anemia because impaired hemoglobin synthesis results in the production of microcytic red blood cells [15]. Given the substantial burden of maternal anemia and its adverse consequences, population-specific data are needed to better understand the relationship between serum ferritin and hemoglobin concentrations during pregnancy [16]. Therefore, this cross-sectional, laboratory-based study was conducted to examine the relationship between serum ferritin and hemoglobin levels among pregnant women and to generate local evidence regarding iron status during pregnancy.

Although the biological relationship between hemoglobin concentration and iron stores is well established, evidence quantifying the strength of this association and identifying women with depleted iron stores despite normal hemoglobin levels remains limited in Pakistan.

Novelty of the study

Recent studies from Pakistan have primarily focused on regional differences, the prevalence of anemia, or comparisons of hematological and biochemical parameters between anemic and non-anemic pregnant women [11,17]. However, evidence evaluating the relationship between serum ferritin and hemoglobin concentrations and the potential utility of serum ferritin in identifying early iron deficiency remains limited. Therefore, this study aimed to address this gap by examining the association between serum ferritin and hemoglobin levels and evaluating the potential of serum ferritin as an early screening marker for iron deficiency among pregnant women in Pakistan.

The novelty of the present study lies in three key aspects. First, it quantifies the strength of the association between serum ferritin and hemoglobin concentrations using effect size measures. Second, it identifies pregnant women with depleted iron stores despite having normal hemoglobin concentrations, thereby highlighting the presence of early iron deficiency that may not be detected by hemoglobin measurement alone. Third, it evaluates the potential clinical utility of serum ferritin as an early screening marker for iron deficiency in this population.

Objectives

The primary objective of this study was to determine the relationship between serum ferritin and hemoglobin levels among pregnant women. The secondary objectives were to determine the prevalence of iron deficiency and iron-deficiency anemia, estimate the proportion of women with low serum ferritin despite normal hemoglobin concentrations, and assess whether the relationship between serum ferritin and hemoglobin levels varied across pregnancy trimesters.

## Materials and methods

Study design and setting

This prospective, cross-sectional, laboratory-based study was conducted at the antenatal care clinic of Combined Military Hospital (CMH), Lahore, Pakistan. The study aimed to investigate the relationship between hemoglobin and serum ferritin levels among pregnant women. Eligible pregnant women attending routine antenatal care during the study period were consecutively recruited. Blood samples were collected as part of routine antenatal investigations and processed according to the hospital's standard laboratory protocols.

Sampling procedure

A non-probability convenience sampling technique was used to recruit participants for this study. Pregnant women attending routine antenatal care at the selected healthcare facility during the study period were consecutively approached for participation. A total of 560 pregnant women were screened for eligibility. Of these, 38 were excluded because they did not meet the inclusion criteria or met one or more exclusion criteria. The final analysis included 522 participants. The sample size was determined based on feasibility and the availability of eligible participants during the study period and was considered sufficient to provide adequate statistical power for comparative and regression analyses.

Study population

The eligibility criteria for study participation are summarized in Table 1.

**Table 1:** Inclusion and exclusion criteria Note: Participants with stable non-iron-related chronic conditions, such as well-controlled chronic (pre-existing) essential hypertension, treated hypothyroidism, and other physician-diagnosed long-term conditions not expected to significantly affect iron metabolism, were eligible for inclusion. Women with gestational hypertension were excluded.

Inclusion criteria	Exclusion criteria
Pregnant women aged ≥18 years	Multiple gestation (e.g., twin pregnancy)
Women in the second trimester	Preeclampsia
Women in the third trimester	Gestational hypertension
Attending the antenatal clinic during the study period	Gestational diabetes mellitus
Provided informed consent and blood sample	Chronic kidney disease
Complete laboratory data available	Known hemoglobinopathies
	Active inflammatory diseases
	Acute infection
	Refusal to provide a blood sample
	Incomplete laboratory investigations

Data collection

Demographics and Clinical Information

Demographic and clinical data were collected using a structured proforma that included age, gestational age, parity, and relevant medical and obstetric history. These variables were included in the descriptive analysis and considered as potential covariates in the statistical analyses, where appropriate. Chronic diseases were documented based on participants' medical history and self-reported information obtained during the antenatal evaluation. Information on oral iron supplementation was also collected using the structured proforma. Participants were classified as regular users if they reported taking oral iron supplements as prescribed during pregnancy, irregular users if they reported inconsistent or occasional use, and non-users if they reported no use of oral iron supplements during pregnancy.

Laboratory assessment

Venous blood samples (5 mL) were collected from each participant using a standard aseptic technique. Blood samples for complete blood count (CBC) analysis were collected into EDTA tubes and analyzed using an automated hematology analyzer to determine hemoglobin concentration and red blood cell indices. CBC parameters were evaluated to identify disproportionate microcytosis, which may not always correspond to the severity of anemia. Clinical history and hematological findings were used to exclude participants with a known history of hemoglobinopathies. Although these assessments excluded participants with known hemoglobinopathies, hemoglobin electrophoresis was not routinely performed, representing a limitation of the study.

For serum ferritin analysis, venous blood was collected into serum separator tubes (SSTs), allowed to clot at room temperature for approximately 20-30 minutes, and centrifuged at approximately 3,000 rpm for 10-15 minutes to separate the serum. Serum samples were analyzed immediately after processing in accordance with the hospital laboratory's routine workflow. Serum ferritin concentrations were measured according to the hospital laboratory's standard operating procedures. Internal quality control procedures and instrument calibration were performed to ensure the accuracy, consistency, and reproducibility of the measurements. The assay methodology used in the hospital laboratory is consistent with validated ferritin immunoassays that have demonstrated good agreement and low systematic variability in previous methodological studies [18,19]. All analyses were performed under standardized laboratory conditions using standardized calibration procedures.

A serum ferritin cutoff of <30 µg/L was selected based on recommendations from the American College of Obstetricians and Gynecologists, reflecting the increased iron requirements and physiological hemodilution that occur during pregnancy [20,21]. This cutoff was used because it is a widely accepted threshold for identifying iron deficiency during pregnancy and improves the detection of early iron depletion before the development of overt anemia. However, additional biomarkers of iron status and inflammation, including transferrin saturation, C-reactive protein (CRP), and procalcitonin, were not routinely available and were therefore not included in the present study. Anemia severity was classified according to hemoglobin concentration as normal (≥11.0 g/dL), mild (9.0-10.9 g/dL), moderate (7.0-8.9 g/dL), or severe (<7.0 g/dL) [22].

Outcomes

Primary Outcome

The primary aim of this study was to assess the relationship between serum ferritin and hemoglobin levels to better understand the association between iron stores and anemia during pregnancy.

Secondary Outcomes

The secondary outcomes of this study were the prevalence of iron deficiency among pregnant women, defined as a serum ferritin concentration <30 µg/L, and the prevalence of iron-deficiency anemia, defined as the coexistence of low serum ferritin and low hemoglobin concentrations. In addition, the study aimed to determine the prevalence of subclinical iron deficiency, defined as depleted iron stores despite normal hemoglobin concentrations. Identifying these early manifestations of iron deficiency may facilitate the recognition of women at increased risk of developing anemia later in pregnancy and support the timely implementation of appropriate nutritional or therapeutic interventions.

Statistical analysis

Data were analyzed using IBM SPSS Statistics for Windows, Version 26.0 (Released 2018; IBM Corp., Armonk, NY, USA). Descriptive statistics were used to summarize the demographic and clinical characteristics of the participants. Categorical variables were presented as frequencies and percentages. The association between anemia status and ferritin status was assessed using the chi-square test, with Cramer's V reported as the measure of effect size. Independent-samples Student's t-tests were used to compare continuous hematological parameters, including hemoglobin concentration, serum ferritin concentration, and MCV, between women with and without anemia. Cohen's d was calculated to quantify the effect size of these comparisons. Binary logistic regression analysis was performed to identify factors associated with anemia, and the results were reported as odds ratios (ORs) with 95% confidence intervals (CIs). For the regression analyses, anemia status was coded as 1 (anemia; hemoglobin < 11.0 g/dL) and 0 (no anemia; hemoglobin ≥ 11.0 g/dL), whereas iron deficiency was coded as 1 (serum ferritin <30 µg/L) and 0 (serum ferritin ≥30 µg/L). A two-sided p-value < 0.05 was considered statistically significant.

Ethical considerations

This study was conducted in accordance with the principles of the Declaration of Helsinki and was approved by the Institutional Review Board of Combined Military Hospital (Approval No. CMH-IRB-ERC-OBG-2025-017). All participants were informed about the purpose of the study, the study procedures, the potential risks and benefits, and the informed consent process before providing written informed consent. Participation was voluntary, and participants were free to withdraw from the study at any time without affecting their medical care. Participant confidentiality was maintained through the use of study identification codes, and all data were stored securely. Blood samples were collected using standard aseptic techniques. Participants diagnosed with iron deficiency or anemia were referred to their respective healthcare providers for appropriate evaluation and management.

## Results

The sociodemographic and clinical characteristics of the 522 participants are presented in Table 2. The largest age group was 25-29 years (n = 182, 34.9%), followed by 20-24 years (n = 130, 24.9%). Participants were equally distributed between the second trimester (n = 261, 50.0%) and the third trimester (n = 261, 50.0%). Most participants were multigravida (n = 235, 45.0%), had completed secondary education (n = 235, 45.0%), were housewives (n = 313, 60.0%), and belonged to the middle socioeconomic group (n = 235, 45.0%). Among the 313 women with at least one previous pregnancy, 157 (50.2%) had an interpregnancy interval of <2 years, whereas 156 (49.8%) had an interval of ≥2 years. Regular oral iron supplementation was reported by 183 participants (35.1%), while 183 of the 444 participants (41.2%) with available data reported a history of anemia. Overall, the study population consisted predominantly of young pregnant women, with a high prevalence of self-reported anemia and relatively low adherence to regular oral iron supplementation.

**Table 2:** Sociodemographic and clinical characteristics of study participants (N = 522) Note: Inter-pregnancy interval was assessed only among women with at least one previous pregnancy (multigravida and grand multigravida participants).

Variable	n	%
Age group		
18-19 years	26	5.0
20-24 years	130	24.9
25-29 years	182	34.9
30-34 years	104	19.9
≥35 years	80	15.3
Gestational age		
2nd trimester	261	50.0
3rd trimester	261	50.0
Parity		
Primigravida	209	40.0
Multigravida	235	45.0
Grand multigravida	78	14.9
Educational status		
No formal education	52	10.0
Primary	105	20.1
Secondary	235	45.0
Intermediate	130	24.9
Socioeconomic status		
Low	183	35.1
Middle	235	45.0
High	104	19.9
Occupation		
Housewife	313	60.0
Employed	104	19.9
Student	53	10.2
Self-employed	52	10.0
Inter-pregnancy Interval (n = 313)		
<2 years	157	50.2
≥2 years	156	49.8
Oral iron supplement use		
Regular	183	35.1
Irregular	209	40.0
None	130	24.9
History of anemia (n = 444)		
Yes	183	41.2
No	261	58.8
Chronic conditions		
Yes	104	19.9
No	418	80.1

Table 3 presents the hematological profile of the study participants. The mean serum ferritin concentration was 28.56 µg/L (SD = 30.09), with a median of 18.90 µg/L. The higher mean than median, together with the large standard deviation, suggests a positively skewed distribution of serum ferritin concentrations. The mean hemoglobin concentration was 11.03 g/dL (SD = 1.63), with a median of 11.20 g/dL, indicating that the average hemoglobin level was close to the diagnostic threshold for anemia.

**Table 3:** Hematological profile of the study participants Note: Ferritin distribution was positively skewed (skewness = 2.23); therefore, median and interquartile range are reported. MCV: mean corpuscular volume.

Variable	M	SD	Median	Min	Max	IQR
Ferritin (µg/L)	28.56	30.09	18.90	2.00	145.00	24.00
Hemoglobin (g/dL)	11.03	1.63	11.20	6.00	14.50	2.20
MCV (fL)	81.51	8.52	81.60	65.00	100.00	12.00

The distribution of hematological categories among the 522 participants is presented in Table 4. Iron deficiency was identified in 209 participants (40.0%), whereas 313 (60.0%) were iron-replete. Anemia, defined as a hemoglobin concentration <11 g/dL, was present in 235 participants (45.0%), while 287 (55.0%) had normal hemoglobin concentrations. Microcytosis, defined by a reduced MCV, was observed in 156 participants (29.9%), whereas 366 participants (70.1%) had normal MCV values. Overall, the findings indicate a substantial prevalence of iron deficiency, anemia, and microcytosis among the study population.

**Table 4:** Distribution of hematological categories (N = 522) Note: Anemia is defined as hemoglobin < 11 g/dL. Serum ferritin < 30 µg/L indicates absolute iron deficiency. Percentages are based on N = 522. MCV: mean corpuscular volume.

Category	n	%
Ferritin status		
Iron-replete (≥30 µg/L)	313	60.0
Iron-deficient (<30 µg/L)	209	40.0
Hemoglobin status		
Normal (≥11 g/dL)	287	55.0
Deficit (<11 g/dL)	235	45.0
MCV status		
Normal (≥80 fL)	366	70.1
Microcytic (<80 fL)	156	29.9
Anemia status		
No anemia	287	55.0
Anemia	235	45.0

Table 5 compares hematological parameters between women with and without anemia. All parameters differed significantly between the two groups. Women without anemia had significantly higher mean hemoglobin concentrations (12.23 ± 0.83 g/dL) than women with anemia (9.57 ± 1.06 g/dL), with a mean difference of 2.66 g/dL (95% CI: 2.46-2.86, p < 0.001) and a very large effect size (Cohen's d = 2.83). Similarly, mean serum ferritin concentrations were significantly higher among women without anemia, with a mean difference of 25.32 µg/L (95% CI: 20.60-30.04, p < 0.001) and a large effect size (Cohen's d = 0.93), indicating substantially greater iron stores than in women with anemia. MCV was also significantly higher in women without anemia (85.60 ± 7.36 fL) than in those with anemia (76.52 ± 7.07 fL), with a mean difference of 9.08 fL (95% CI: 7.82-10.34, p < 0.001) and a large effect size (Cohen's d = 1.25). Overall, women with anemia had significantly lower hemoglobin concentrations, serum ferritin concentrations, and MCV values than women without anemia. The observed differences were statistically significant and associated with large to very large effect sizes, indicating clinically meaningful differences between the two groups.

**Table 5:** Comparison of hematological parameters between women with and without anemia using independent samples Student’s t-test and effect size estimation Note: Independent samples t-test was used. Confidence intervals represent mean differences between groups. All markers were significantly higher in non-anemic women. Cohen’s d indicates effect size. MCV: mean corpuscular volume.

Variable	No anemia, M (SD)	Anemia, M (SD)	Mean difference	95% CI of difference	Statistical test	t (520)	p-value	Cohen's d
Hemoglobin (g/dL)	12.23 (0.83)	9.57 (1.06)	2.66	2.46, 2.86	Independent samples Student’s t-test	32.19	<0.001	2.83
Ferritin (µg/L)	39.96 (33.17)	14.64 (17.82)	25.32	20.60, 30.04	Independent samples Student’s t-test	10.53	<0.001	0.93
MCV (fL)	85.60 (7.36)	76.52 (7.07)	9.08	7.82, 10.34	Independent samples Student’s t-test	14.27	<0.001	1.25

Table 6 presents the association between anemia status and ferritin status among the 522 study participants. Among women with anemia (n = 235), 42 (17.9%) were iron-deficient, whereas 193 (82.1%) were iron-replete. In contrast, among women without anemia (n = 287), 167 (58.2%) were iron-deficient and 120 (41.8%) were iron-replete. Overall, 209 participants (40.0%) were classified as iron-deficient, whereas 313 (60.0%) were iron-replete. The chi-square test demonstrated a statistically significant association between anemia status and ferritin status (χ²(1, N = 522) = 87.47, p < 0.001), with a moderate effect size (Cramer's V = 0.409). These findings indicate a significant association between ferritin status and anemia status in the study population.

**Table 6:** Association between anemia status and ferritin status in the study sample (N = 522) Note: Values are frequencies. Ferritin status was classified as iron-deficient (<30 µg/L) or iron-replete (≥30 µg/L). A chi-square test of independence showed a significant association between anemia status and ferritin status, χ²(1, N = 522) = 87.47, p < 0.001. Effect size is reported using Cramer's V.

Anemia status	Ferritin status		Total	x^2^ (df = 1)	p-value	Cramer’s V
	Iron-deficient (<30 µg/L)	Iron-replete (≥30 µg/L)				
Anemic	42 (17.9%)	193 (82.1%)	235 (100.0%)			
Non-anemic	167 (58.2%)	120 (41.8%)	287 (100.0%)			
Total	209 (40.0%)	313 (60.0%)	522 (100.0%)	87.47	<0.001	0.409

Table 7 presents the predictors of anemia, the analysis of pre-anemic risk, and subgroup analyses among women without anemia. The results of the binary logistic regression analysis (Section A) indicated that serum ferritin concentration and MCV were both significant predictors of anemia. Higher serum ferritin concentrations (OR = 0.95, 95% CI: 0.94-0.97, p < 0.001) and higher MCV values (OR = 0.87, 95% CI: 0.85-0.90, p < 0.001) were independently associated with lower odds of anemia. Among women without anemia (Section B), 35 participants (12.2%) had low serum ferritin concentrations despite normal hemoglobin levels, indicating subclinical iron deficiency and representing a subgroup at increased risk of developing anemia. In the logistic regression model evaluating predictors of pre-anemic status (Section C), increasing age was associated with lower odds of pre-anemia (OR = 0.61, p = 0.006), whereas oral iron supplement use was associated with higher odds (OR = 2.04, p = 0.003). This finding may reflect reverse causation, whereby women at greater risk of iron deficiency were more likely to use iron supplements. Parity and gestational age were not significantly associated with pre-anemic status. Section D compares hematological parameters among women without anemia according to pregnancy trimester. No significant differences were observed in serum ferritin concentration, hemoglobin concentration, or MCV between the second and third trimesters (all p > 0.05), suggesting relative stability of these hematological parameters during the later stages of pregnancy in this subgroup.

**Table 7:** Predictors of anemia and pre-anemic risk Note: Logistic regression used the enter method. Odds ratios (ORs) represent the change in odds for a one-unit increase in the predictor. Trimester comparisons were conducted using the Mann-Whitney U test. No statistically significant differences were observed between trimesters. MCV: mean corpuscular volume, Hb: hemoglobin.

Section	Predictor	B	SE	OR/n/mean rank	95% CI/%/mean rank	U/Z	p-value
A. Logistic regression predicting anemia (N = 522)	Ferritin	-0.048	0.008	0.95	0.94, 0.97		<0.001
	MCV	-0.136	0.017	0.87	0.85, 0.90		<0.001
	Constant	11.883	1.353				<0.001
B. Pre-anemic group distribution (n = 287)	Normal ferritin + normal Hb			252	87.8%		
	Low ferritin + normal Hb			35	12.2%		
C. Logistic regression predicting pre-anemic status (n = 287)	Age group	-0.496	0.180	0.61	0.43, 0.87		0.006
	Parity	-0.476	0.291	0.62	0.35, 1.10		0.102
	Iron supplement use	0.715	0.244	2.04	1.27, 3.30		0.003
	Gestational age	-0.368	0.384	0.69	0.33, 1.47		0.338
	Constant	-0.473	1.017				0.642
D. Trimester comparison among non-anemic women (n = 287)	Ferritin			137.80	150.15	9410/-1.26	0.208
	Hemoglobin			147.45	140.57	9802.50/-0.70	0.482
	MCV			141.83	146.15	9986/-0.44	0.659

**Figure 1:**
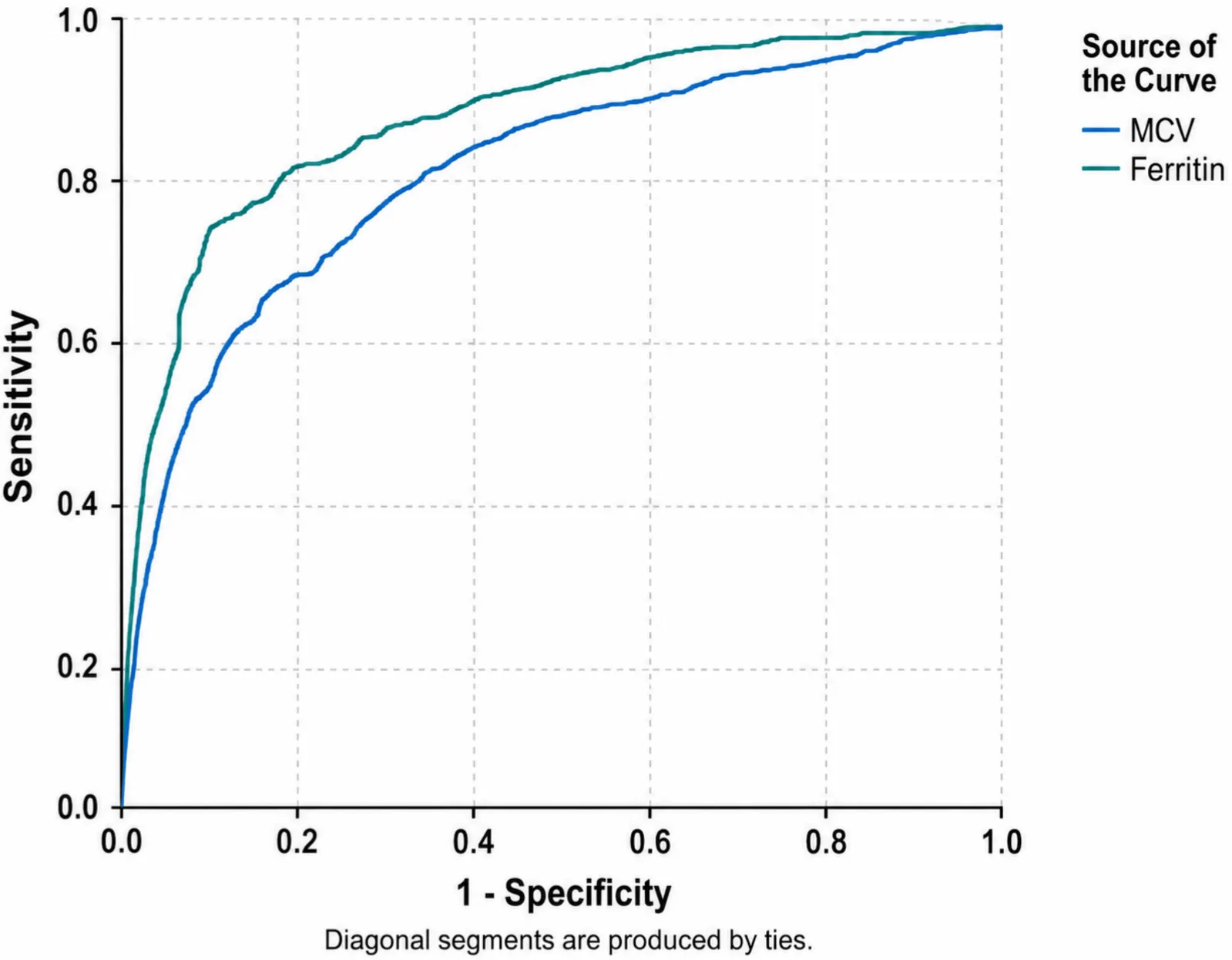
Receiver operating characteristic (ROC) curves for ferritin and mean corpuscular volume (MCV) in detecting anemia

Figure 1 presents the ROC curves for serum ferritin and MCV in discriminating anemia and the determination of sample-derived cutoff values using the Youden Index (J = Sensitivity + Specificity − 1). The Youden Index was calculated for all ROC coordinates, and the cutoff corresponding to the highest J value was selected as the optimal threshold. For serum ferritin, the optimal cutoff identified in this dataset was 17.0 µg/L (sensitivity = 81%, specificity = 83%, J = 0.64), whereas the optimal MCV cutoff was 79.5 fL (sensitivity = 77%, specificity = 82%, J = 0.58). Both markers demonstrated good discriminative performance, as reflected by their areas under the ROC curve (AUC). Serum ferritin showed slightly greater diagnostic accuracy (AUC = 0.85, 95% CI: 0.82-0.88) than MCV (AUC = 0.81, 95% CI: 0.77-0.85). ROC curves were generated with the test direction reversed so that lower values corresponded to a higher likelihood of anemia, with sensitivity plotted against 1 − specificity. Overall, the ROC curve for serum ferritin consistently lay above that of MCV, indicating slightly superior discriminative performance, although both markers demonstrated clinically useful diagnostic performance.

## Discussion

This study showed that pregnant women with anemia had significantly lower serum ferritin levels than non-anemic women, highlighting the important role of iron deficiency in the development of gestational anemia. Our study demonstrated low ferritin levels, borderline hemoglobin, and a lower-normal MCV, indicative of early or evolving iron deficiency. Past research has found similar results, with anemia and iron deficiency prevalence of up to 60.7% under similar diagnostic thresholds. This underscores the high burden of iron deficiency among such populations [23,24].

One of the main results of the current research is that the prevalence of iron deficiency and anemia is relatively high and constitutes an important public health concern in the research population. The prevalence of microcytosis also confirms iron deficiency as the main underlying pathology, in line with earlier research showing a high rate of latent iron deficiency and microcytic features as the most frequent hematological feature. Other causes, such as thalassemia trait, may also contribute to microcytosis. However, because hemoglobin electrophoresis was not performed, thalassemia trait could not be excluded in the present study. Furthermore, in the presence of iron deficiency, the diagnosis of thalassemia trait can only be reliably established after iron deficiency has been corrected. These findings highlight persistent gaps in nutritional status compared to regional studies. Interestingly, a significant proportion of respondents reported irregular or no iron supplementation, which could partially explain the hematological deficiencies [25,26]. The prevalence of microcytosis was relatively high in this study and should be interpreted with caution. Although iron deficiency is a probable cause, this observation might also reflect the underlying burden of hemoglobinopathies, such as thalassemia trait, which are relatively common among South Asian groups. Since hemoglobin electrophoresis was not carried out regularly, the role of these conditions cannot be ruled out. Thus, the occurrence of microcytosis is high and should be viewed with caution, and may not necessarily be a case of iron deficiency.

Hemoglobin, ferritin, and MCV differ significantly between anemic and non-anemic women, indicating clinically significant differences between the two groups. Lower hemoglobin levels, iron-depleted stores, and more pronounced microcytosis were evident in anemic women, consistent with previous studies reporting similar hematological profiles [27,28]. The results support an association between serum ferritin levels and anemia in this study population. However, serum ferritin should be interpreted with caution when used in isolation, as it may be influenced by inflammation and other clinical conditions.

The logistic regression analysis identified serum ferritin and MCV as significant independent factors associated with anemia. The description of the pre-anemic subgroup, i.e., women whose ferritin is low but hemoglobin is normal, is especially significant, as it highlights early iron deficiency that cannot be detected by hemoglobin screening [27]. The pre-anemic status was significantly associated with younger age, suggesting that younger age is more susceptible to early iron depletion, a finding also reported in previous studies [29].

Interestingly, iron supplementation was associated with increased odds of pre-anemia, which may also be due to confounding by indication, i.e., women already at risk are more likely to receive supplementation. The observation is consistent with past studies showing that supplementation is frequently initiated in response to perceived or suspected deficiency, rather than as a preventive measure [30]. Gestational age and parity did not have a significant effect, implying that early iron depletion may be more influenced by underlying nutritional status than by obstetric factors [31,32].

Moreover, there were no significant changes in ferritin, hemoglobin, or MCV between the second and third trimesters in non-anemic women, suggesting these parameters remained relatively stable in the absence of an apparent deficiency. This has been observed before in other studies, which have found that iron status does not decrease much in later pregnancy, provided there are adequate reserves [33].

The analysis of the ROC curve also indicates the clinical value of ferritin, which has better discriminative capacity than MCV for detecting anemia (AUC 0.849 vs. 0.810). Although both markers demonstrated good discriminative performance, serum ferritin showed higher sensitivity and specificity than MCV, suggesting its potential utility as an adjunct marker for assessing iron status.

Overall, these findings suggest that serum ferritin may provide additional information for assessing iron status during pregnancy, particularly among women with normal hemoglobin levels but depleted iron stores. However, prospective studies are needed to determine its clinical utility and role in antenatal screening.

Limitations

These findings have several limitations that should be noted when interpreting them. First, the cross-sectional design limits the study's causal interpretation because it cannot establish temporal relationships between declining ferritin levels and the onset of anemia. Second, the single-center design and use of convenience sampling may have introduced selection bias and limit the generalizability of the findings to the broader population of pregnant women. Also, details regarding the ferritin assay platform and manufacturer were not available, which may limit the reproducibility of the laboratory methodology. Third, serum ferritin is an acute-phase reactant. It could be affected by subclinical inflammation, but inflammatory markers, including C-reactive protein, were not assessed, which may have led to misclassification of iron status. Additionally, subgroup analyses of isolated iron deficiency and iron deficiency anemia were not performed, limiting further characterization of these clinically relevant groups. Also, the main logistic regression model for anemia did not consider age, parity, gestational age, or iron supplementation as potential confounders. However, they were incorporated into the pre-anemic regression model and could have affected the results. Fourth, hemoglobin electrophoresis was not a standard procedure, so unidentified hemoglobinopathies cannot be excluded. Furthermore, because inflammatory biomarkers and additional indicators of iron metabolism, such as transferrin saturation, were not available, functional iron deficiency could not be distinguished from absolute iron deficiency.

Also, dietary intake and adherence to iron supplementation were not measured or quantified, which limited understanding of behavioral factors contributing to iron deficiency. Also, iron-deficiency anemia and thalassemia trait cannot be distinguished by the absence of hemoglobin electrophoresis. Lastly, serum folate and vitamin B12 levels were not routinely measured; therefore, concurrent deficiencies could not be evaluated. Consequently, the possibility of normocytic anemia resulting from combined iron, folate, or vitamin B12 deficiency cannot be excluded.

Future directions

Longitudinal study designs should be used in future studies to monitor dynamic changes in ferritin and hemoglobin during pregnancy and to determine time-based relationships. The accuracy of ferritin interpretation can be enhanced by including inflammatory markers such as C-reactive protein. Interventional studies assessing the efficacy of ferritin-guided supplementation strategies to mitigate the occurrence of anemia are also required. Generalizability would be improved by increasing the number of studies across various centers and populations. Moreover, the combination of dietary assessments and adherence measures might offer a more detailed insight into the potential modifiable risk factors. Future studies should evaluate the potential clinical value of early pregnancy ferritin assessment and targeted interventions for women with depleted iron stores but normal hemoglobin levels.

## Conclusions

In conclusion, this study found that pregnant women with anemia had significantly lower serum ferritin levels than non-anemic women, and a substantial proportion of women with normal hemoglobin had low serum ferritin levels, suggesting that iron deficiency may be present before the development of overt anemia. These findings support the potential value of serum ferritin as an adjunct marker for assessing iron status during pregnancy. However, further prospective studies are needed to validate these findings and determine their clinical applicability.

## References

[1] Hira Asrar, Ashfaq M, Ali SS (2025). Prevalence of anemia in pregnant women admitted in antenatal wards in Faisalabad 2025, Punjab, Pakistan. J Med Health Sci Rev.

[2] Qiao Y, Di J, Yin L, Huang A, Zhao W, Hu H, Chen S (2024). Prevalence and influencing factors of anemia among pregnant women across first, second and third trimesters of pregnancy in monitoring areas, from 2016 to 2020: a population-based multi-center cohort study. BMC Public Health.

[3] Ogu RN, Ikimalo J (2018). The impact of hematinics supplementation during pregnancy on maternal anemia and perinatal outcome among parturients in Southern Nigeria - a prospective study. J Gynecol Womens Health.

[4] James AH (2021). Iron deficiency anemia in pregnancy. Obstet Gynecol.

[5] Veena Sangkhae, Fisher AL, Ganz T, Nemeth E (2023). Iron homeostasis during pregnancy: maternal, placental, and fetal regulatory mechanisms. Annu Rev Nutr.

[6] Hamid K, Ajaz M, Akhter M, Khan SA, Sadiq MS, Aitazaz F (2022). Prevalence of anemia in pregnant women. Pak J Physiol.

[7] Soofi S, Khan GN, Sadiq K (2017). Prevalence and possible factors associated with anaemia, and vitamin B ((12)) and folate deficiencies in women of reproductive age in Pakistan: analysis of national-level secondary survey data. BMJ Open.

[8] Daru J, Allotey J, Peña-Rosas JP (2017). Serum ferritin thresholds for the diagnosis of iron deficiency in pregnancy: a systematic review. Transfus Med.

[9] Jung DW, Park JH, Kim DH (2017). Association between serum ferritin and hemoglobin levels and bone health in Korean adolescents: A nationwide population-based study. Medicine (Baltimore).

[10] Fu S, Chen J, Liu B (2020). Systemic inflammation modulates the ability of serum ferritin to predict all-cause and cardiovascular mortality in peritoneal dialysis patients. BMC Nephrol.

[11] Asghar S, Azmat S, Rasheed S, Javaid MF, Maqbool R, Anwar M (2024). Evaluation of iron deficiency anemia in pregnancy. Pak J Health Sci.

[12] Mahmood A, Parveen A, Arif A, Shabbir A, Khalid S, Ijaz A (2024). The effect of maternal anemia and iron deficiency on fetal erythropoiesis: comparison between serum erythropoietin, hemoglobin and ferritin levels in mothers and newborns. J Popul Ther Clin Pharmacol.

[13] Quezada-Pinedo HG, Cassel F, Muckenthaler MU (2022). Ethnic differences in adverse iron status in early pregnancy: a cross-sectional population-based study. J Nutr Sci.

[14] Chibanda Y, Brookes M, Churchill D, Al-Hassi H (2023). The ferritin, hepcidin and cytokines link in the diagnosis of iron deficiency anaemia during pregnancy: a review. Int J Mol Sci.

[15] Cappellini MD, Musallam KM, Taher AT (2020). Iron deficiency anaemia revisited. J Intern Med.

[16] Davidson EM, Scoullar MJ, Peach E (2023). Quantifying differences in iron deficiency-attributable anemia during pregnancy and postpartum. Cell Rep Med.

[17] Rehan S, Khattak SN, Khattak MI, Hadi S, Rehman S, Sami Z, Ahmad K (2024). Regional disparities in maternal blood parameters during pregnancy: a comparative analysis across four provinces of Pakistan. J Islamabad Med Dent Coll.

[18] Dahman LS, Sumaily KM, Sabi EM (2022). A comparative study for measuring serum ferritin levels with three different laboratory methods: enzyme-linked immunosorbent assay versus Cobas e411 and Cobas integra 400 methods. Diagnostics (Basel).

[19] Garcia-Casal MN, Peña-Rosas JP, Urrechaga E, Escanero JF, Huo J, Martinez RX, Lopez-Perez L (2018). Performance and comparability of laboratory methods for measuring ferritin concentrations in human serum or plasma: a systematic review and meta-analysis. PLoS One.

[20] Lewkowitz AK, Tuuli MG (2023). Identifying and treating iron deficiency anemia in pregnancy. Hematology Am Soc Hematol Educ Program.

[21] Khan FF, Jahan MS, Nahar MI, Parvin R, Eva NJ, Ahmed N, Nesa MK (2025). Correlation between red cell indices and serum ferritin level in pregnant women with latent iron deficiency. Int J Reprod Contracept Obstet Gynecol.

[22] Baig-Ansari N, Badruddin SH, Karmaliani R (2008). Anemia prevalence and risk factors in pregnant women in an urban area of Pakistan. Food Nutr Bull.

[23] Kolarš B, Mijatović Jovin V, Živanović N, Minaković I, Gvozdenović N, Dickov Kokeza I, Lesjak M (2025). Iron deficiency and iron deficiency anemia: a comprehensive overview of established and emerging concepts. Pharmaceuticals (Basel).

[24] Noghabaei G, Arab M, Payami S, Ghavami B, Nouri B, Parkhideh R (2024). Frequency of anemia/IDA and associated risk factors among working women of a medical center in Tehran, Iran: a cross-sectional study. Indian J Community Med.

[25] Rana SS, Nayyar A, Javed R, Mohsin M, Salman S, Javed A (2026). Detection of latent iron deficiency and thalassemia trait with red cell indices and peripheral blood smear. Med Forum Mon.

[26] Guseibat TA, Said MM (2020). A comparative study on the prevalence of microcytic anemia and associated age of anemic patients from Benghazi city. J Pure Appl Sci.

[27] Anwar Z, Abid Z, Ghazenfer T (2020). Biochemical and hematological profile of anemic and non-anemic pregnant women. J Dow Univ Health Sci.

[28] Hirosawa T, Hayashi A, Harada Y, Shimizu T (2022). The clinical and biological manifestations in women with iron deficiency without anemia compared to iron deficiency anemia in a general internal medicine setting: a retrospective cohort study. Int J Gen Med.

[29] Sekhar DL, Murray-Kolb LE, Kunselman AR, Weisman CS, Paul IM (2016). Differences in risk factors for anemia between adolescent and adult women. J Womens Health (Larchmt).

[30] Patel N, Silvey SG, Arora P, Feldman GM (2024). Optimal oral iron therapy for iron deficiency anemia among US Veterans. JAMA Netw Open.

[31] Lipoeto NI, Masrul Masrul, Nindrea RD (2020). Nutritional contributors to maternal anemia in Indonesia: chronic energy deficiency and micronutrients. Asia Pac J Clin Nutr.

[32] Zhou H, Lu Y, Luo J, Pan B, Zhao Q, Chen M, Ma ZF (2025). Maternal iron deficiency assessed by serum ferritin and birth outcomes in mainland China. Sci Rep.

[33] Rahmati N, Naeiji Z, Ashrafivand S, Toryal M (2025). Prevalence of iron deficiency in non-anemic pregnant women during the first trimester: a prospective cohort study. Jundishapur J Chronic Dis Care.

